# Neural correlates of cognitive aging during the perception of facial age: the role of relatively distant and local texture information

**DOI:** 10.3389/fpsyg.2015.01420

**Published:** 2015-09-23

**Authors:** Jessica Komes, Stefan R. Schweinberger, Holger Wiese

**Affiliations:** ^1^DFG Research Unit Person Perception, Friedrich Schiller University of JenaJena, Germany; ^2^Department of Psychology, Durham UniversityDurham, UK

**Keywords:** face perception, N170, inversion effect, aging, own-age bias

## Abstract

Previous event-related potential (ERP) research revealed that older relative to younger adults show reduced inversion effects in the N170 (with more negative amplitudes for inverted than upright faces), suggestive of impairments in face perception. However, as these studies used young to middle-aged faces only, this finding may reflect preferential processing of own- relative to other-age faces rather than age-related decline. We conducted an ERP study in which young and older participants categorized young and old upright or inverted faces by age. Stimuli were presented either unfiltered or low-pass filtered at 30, 20, or 10 cycles per image (CPI). Response times revealed larger inversion effects, with slower responses for inverted faces, for young faces in young participants. Older participants did not show a corresponding effect. ERPs yielded a trend toward reduced N170 inversion effects in older relative to younger adults independent of face age. Moreover, larger inversion effects for young relative to old faces were detected, and filtering resulted in smaller N170 amplitudes. The reduced N170 inversion effect in older adults may reflect age-related changes in neural correlates of face perception. A smaller N170 inversion effect for old faces may indicate that facial changes with age hamper early face perception stages.

## Introduction

Aging leads to a number of changes in humans. Two of these changes, which are often considered as particularly salient, relate to a decline in perceptual and cognitive abilities and to age-related changes in facial appearance. As we grow older, attention, working and episodic memory, language processing and executive functions undergo age-related modifications, often (but not always) resulting in less efficient performance in older relative to younger adults (see e.g., Craik and Salthouse, 2008). In addition, several aspects of face perception and memory have been described to become less efficient over the adult lifespan (Hildebrandt et al., 2010, 2011). At the same time, aging typically results in characteristic changes of the texture (e.g., wrinkling) and coloration of the skin's surface, as well as the shape of the face (e.g., Burt and Perrett, 1995), which allow the viewer to roughly estimate the age of another person. The research presented in this paper is at the intersection of these two types of age-related changes, as we examined the perception of facial age in young and older adult participants. More specifically, we were interested in whether the perception of facial age cues would be modulated by the age of the perceiver.

As noted above, a number of different characteristics allow the perception of facial age. One of these characteristics appears to be related to configural information, a term that is used with slightly different meanings by different authors (for a critical discussion, see Burton et al., 2015). Reviewing literature on face image matching tasks, which are often assumed to capture identity processing (but see e.g., Maurer et al., 2002; Burton, 2013; Burton et al., 2015) distinguish three types of configural processing: (1) the sensitivity to first-order relations, which reflects a basic configuration of features shared by all faces, with two eyes above a nose, which is above a mouth, (2) holistic processing, which refers to the integration of facial features into a Gestalt-like representation, and (3) the sensitivity to second-order relations, which reflects the perception of the detailed spatial layout of, and metric distances between, facial features. It is commonly reported that all types of configural processing are substantially disturbed by face inversion, i.e., the picture-plane rotation of the image by 180° (Yin, 1969), whereas local information is not to the same extent (Maurer et al., 2002; Rossion, 2008), although some authors observed results diverging from this widely accepted view (see e.g., Sekuler et al., 2004). In line with the former findings, it has been suggested that inversion results in a narrowing of the perceptual field, which only allows the analysis of relatively local information, but not the simultaneous processing of information from multiple features distributed over a large space of the face (Rossion, 2009). Following this suggestion, we will assume for the present manuscript that inversion disrupts the simultaneous processing of relatively distant information in faces, and not (or not to the same extent) the processing of relatively local information.

While the studies discussed in the preceding paragraph mostly report results from face matching tasks aimed at examining identity processing, previous research suggests that processing relatively distant information is also important for the perception of facial age. Although one might argue that perceiving relatively local qualities of the skin's texture (such as the presence vs. absence of wrinkles) would suffice for a rough and dichotomous age categorization, previous research demonstrated that deciding whether an adult face is young or old is slowed by face inversion (Wiese et al., 2012a). Moreover, using the composite face paradigm (Young et al., 1987), in which two halves from different faces are combined to form a novel whole face, Hole and George (2011) found that estimating the exact age of the upper half of a composite is systematically biased toward the age of the lower half, indicating an influence of the task-irrelevant part of the face and thus holistic processing during age estimations.

However, evidence for the simultaneous use of information from relatively distant parts of the face during age perception is not as clear-cut as it may seem from the two studies described in the last paragraph. For instance, estimates of the exact age of a face are similarly accurate for upright and inverted faces (George and Hole, 2000). Together with the above-discussed finding of slower age categorization for inverted faces, this finding suggests that age perception is less efficient but similarly accurate (i.e., more time-consuming processing is necessary to reach the same level of accuracy) when information from relatively distant parts is largely absent. Moreover, George and Hole (2000) reduced the availability of skin texture information by low-pass filtering the face images. Again, this manipulation did not lead to any impairment in the accuracy of age estimations. The authors concluded that facial age could be estimated from a number of different and independent cues, and that whichever cues are currently available can be flexibly used. It is unclear, however, whether this is similarly true for the efficiency of age categorizations.

Importantly, face perception depends to some extent on the amount of expertise the viewer has with a particular category of faces. For instance, it has been shown that own-race faces are perceived more holistically than other-race faces (Michel et al., 2006). In addition, advantages in part-based and second-order configural processing for own- relative to other-race faces have been observed (for a review, see Hayward et al., 2013). Moreover, it has been shown that young adults typically have more experience with young relative to older faces, whereas older adults either have balanced experience or a bias toward older faces (e.g., Wiese et al., 2012b). Presumably related to these differences in experience, an own-age advantage has been observed in recognition memory (e.g., Bartlett and Leslie, 1986; Rhodes and Anastasi, 2012; Wiese et al., 2013b), face matching tasks (Macchi Cassia, 2011; Verdichevski and Steeves, 2013), and in age estimations (Moyse and Bredart, 2012; Voelkle et al., 2012). Accordingly, young and older adults seem to perceive young and old adult faces differently. For instance, previous findings of faster ethnicity categorizations of other-relative to own-race faces (Valentine and Endo, 1992) could motivate the prediction that whereas own-age faces are remembered more accurately in a recognition memory task, other-age faces will tend to be processed more efficiently in an age categorization task.

Whereas, the behavioral measures discussed so far can only depict the outcome of a cascade of different processing steps, time-sensitive measures of neural activity may be more suited to track the various sub-stages of stimulus processing. Given their high temporal resolution, event-related potentials (ERPs) appear particularly well-suited for this endeavor. ERPs are voltage changes in the electroencephalogram time-locked to a specific event, such as the presentation of a visual stimulus. ERPs largely reflect current changes at the postsynaptic membrane (Jackson and Bolger, 2014) and thus provide a measure of the brain's neural activity.

ERP studies on face perception have identified a negative occipito-temporal peak at approximately 170 ms after stimulus onset, the so-called N170 component, to be reliantly larger to faces as compared to other objects (Bentin et al., 1996; Eimer, 2011). The N170 is typically assumed to reflect early stages of face perception, related to the detection of a face-like pattern (Schweinberger and Burton, 2003; Amihai et al., 2011), which may correspond to first-order configural processing in terms of Maurer et al. (2002), or structural encoding (see e.g., Eimer, 2011), a term which is derived from the model by Bruce and Young (1986) and which denotes perceptual processes prior to individual face recognition. Moreover, this component is sensitive to a number of the manipulations and facial characteristics discussed above: it has been reported to be (1) increased and delayed for inverted relative to upright faces (reflecting the so-called N170 inversion effect; e.g., Eimer, 2000a; Rossion et al., 2000; Itier and Taylor, 2002), (2) smaller for spatially low-pass filtered relative to full spectrum faces (Goffaux et al., 2003; Halit et al., 2006; but see Holmes et al., 2005), (3) increased for other- relative to own-race faces (e.g., Herrmann et al., 2007; Caharel et al., 2011; Wiese et al., 2014), at least when face category or identity is task-relevant (Wiese, 2013), and (4) larger for old relative to young adult faces (Wiese et al., 2008, 2013c; Wolff et al., 2012):(for a related finding on the frontal P2, or VPP, see Ebner et al., 2011). Interestingly, at least some of the processes underlying N170 seem to be modulated by experience, as larger inversion effects for own- relative to other-race faces have been observed (Vizioli et al., 2010; Caharel et al., 2011; Wiese, 2013).

Moreover, several studies used the N170 to examine age-related changes in face perception. First, generic face sensitivity of N170, with larger amplitudes for faces vs. objects, was found similarly in young and older adults (Gao et al., 2009; Daniel and Bentin, 2012), suggesting preserved neural sensitivity for faces in higher age. Second, smaller N170 inversion effects have been observed in older participants (Gao et al., 2009; Daniel and Bentin, 2012). Finally, the typical lateralization of the N170, with larger amplitudes over the right relative to the left hemisphere (Bentin et al., 1996; Amihai et al., 2011; Eimer, 2011), has been found to be less pronounced in older adults (Pfütze et al., 2002; Gao et al., 2009; Daniel and Bentin, 2012), which may reflect an attempt to compensate for age-related decline (Komes et al., 2014b). Thus, evidence for age-related changes of early face perception on the basis of the N170 is mixed. Whereas, the component's sensitivity to faces seems unchanged, both its lateralization and the N170 inversion effect seem affected by aging. It should be noted, however, that stimulus sets in previous studies showing reduced inversion effects in older adults were dominated by young and mid-aged faces, and that N170 inversion effects have been observed to be larger for own- relative to other-group faces (Vizioli et al., 2010; Wiese, 2013). This may have biased the results, as own-age faces were presented for young but not older participants, and it is thus unclear whether reduced inversion effects in older adults will also occur when old faces are presented.

Finally, ERPs subsequent to the N170 seem to be affected by aging. Whereas, in younger adults a clearly defined positive-going peak, often referred to as the P2, occurs subsequent to N170, this component is clearly reduced in older adults (Wiese et al., 2008; Rousselet et al., 2009). At the same time, effects of face inversion or low-pass filtering on P2 have not been described in older adults. Some authors have associated the P2 with second-order configural processing (Latinus and Taylor, 2006), whereas others suggested that it is related to the distinctiveness of faces (Schulz et al., 2012). Moreover, P2 is strongly affected by spatial attention during face processing tasks (Neumann et al., 2015), and larger for young relative to old faces in both young and older adults (Wiese et al., 2008, 2012b). Overall, for the purpose of examining effects of the participants' age on age perception, an analysis of P2, in addition to N170, seems necessary.

In the present study we asked young and older adult participants to categorize young and older adult faces by age. The faces were presented in upright or inverted orientation as well as in unfiltered or low-pass filtered versions. Our aims were three-fold: First, we wanted to examine the relative importance of processing relatively distant vs. local texture-based information for age categorization. It has been suggested that inversion narrows the perceptual field, disturbing the simultaneous processing of relatively distant parts of the face (Rossion, 2009). Low-pass filtering, in turn, removes wrinkles, and smoothes locally restricted changes in skin coloration, and therefore hinders the processing of local surface texture cues (Kloth et al., 2015). As previous studies demonstrated less efficient age categorization of inverted faces (Wiese et al., 2012a), we were interested in testing whether filtering the images would result in an additional decrease in performance. We used a stepwise filtering approach with increasingly severe cut-off frequencies (unfiltered, 30 CPI, 20 CPI, 10 CPI) to more precisely identify the frequency range informative for age perception. Similarly, at the neural level, we were interested to see how the combination of filtering and inversion, which have been described to have opposite effects on N170 amplitude, would affect ERPs reflecting perceptual processing stages.

Second, we considered that age categorization may be easier for a certain age category (e.g., for other-age vs. own-age faces, or for young vs. old faces) and/or may be modulated by the viewer's experience. If so, any processing advantage for a specific category of faces in one participant group (such as more efficient categorization of old faces in young adults, which would parallel the above-described finding of more efficient categorization of other-race faces) should be absent or even reversed in the other participant group. Previous studies observed a larger N170 inversion effect (with more negative amplitudes for inverted relative to upright faces) for own- relative to other-race faces. If the early perceptual processing of facial age similarly relied on expertise, larger inversion effects for own- relative to other-age faces would be expected.

Finally, and related to this latter point, we tested whether older adults would be less efficient in face perception, and in age categorization specifically. Previous findings of smaller N170 inversion effects in older relative to younger adults may have been related to the use of young face stimuli. If early perceptual processing of facial age was modulated by expertise, using young faces may have resulted in an advantage for young participants. Consequentially, we considered the possibility that previous findings of reduced N170 inversion effects in older adults might not reflect less efficient face processing *per se*, to the extent that older adults would show similar inversion effects for old faces as young adults do for young faces.

## Materials and methods

### Participants

Twenty-four undergraduate students (mean age = 21.5 years, *SD* = 2.0, 16 female) and 24 older participants (mean age = 65.8 years, *SD* = 4.3, 13 female) participated in the study. Older adults were recruited in senior citizen groups and via a press release in a local newspaper, and were reimbursed with 7.50 Euro per hour. All participants were Caucasian, reported to reside in independent living conditions and were right handed according to a modified version of the Edinburgh Handedness Inventory (Oldfield, 1971). None reported psychiatric or neurological disorders or received central acting medication, and all participants reported normal or corrected-to-normal vision. Furthermore, all participants gave written informed consent and the study was approved by the local Faculty ethics committee.

### Stimuli

**Figure 1:**
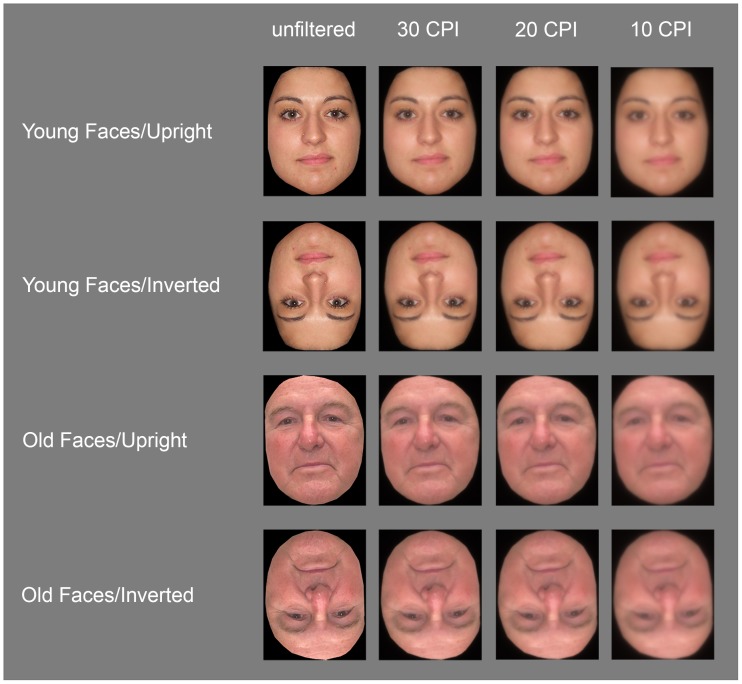
**Examples of the face stimuli used in the present experiment**.

Stimuli consisted of 50 old (mean age = 77.5 years, *SD* = 6.7) and 50 young Caucasian faces (*M* = 22.1 years, *SD* = 2.42), 50% female respectively, and all taken from the CAL/PAL database (Minear and Park, 2004). All pictures displayed front views of neutral faces and were edited in Adobe Photoshop™ to remove all information (hair, clothing, background, etc.) apart from the face, which was subsequently pasted in front of a black background. All stimuli were framed within an area of 170 × 216 pixels (6.0 × 7.6 cm), corresponding to a visual angle of 3.8° × 4.8° at a viewing distance of 90 cm. Images were then filtered with the FourierImage software developed by Risto Näsänen (http://nasanen.info/Software.html) using an exponential low-pass filter with cut-off frequencies set to 30, 20, or 10 cycles per image (CPI). Furthermore, all stimuli were presented in both upright and inverted orientation, as well as in unfiltered and three low-pass filtered versions, resulting in eight images of each individual face (see Figure 1 for stimulus examples).

### Procedure

Participants were seated in a dimly lit, electrically shielded, and sound–attenuated chamber (400A-CT_Special, Industrial Acoustics, Niederkrüchten, Germany) with their heads in a chin rest. Approximate distance between eyes and computer screen was 90 cm. Each experimental session began with a series of practice trials on different stimuli, which were excluded from data analysis. On each trial, a face stimulus was presented for 1000 ms, preceded by a fixation cross for 2000 ms.

The main experiment consisted of five blocks with 160 trials each, i.e., 800 trials in total. All 50 young and 50 old face identities were presented once in each of the eight stimulus versions. Within each block, 10 trials per experimental condition were presented, with a maximum of one repetition of facial identities per block. Blocks were presented in fixed order, and individual stimuli were presented in random order within each block. Participants were instructed to categorize each face according to age as fast as possible and without compromising accuracy. Between each block, participants were allowed a self-timed period of rest. Key assignment was counterbalanced across participants. Mean response times (RT, correct responses only) and accuracy was analyzed.

### ERP recording and analysis

We recorded 32-channel EEG using a BioSemi Active II system (BioSemi, Amsterdam, Netherlands). The active sintered Ag/Ag-Cl-electrodes were mounted in an elastic cap. EEG was recorded continuously from Fz, Cz, Pz, Iz, FP1, FP2, F3, F4, C3, C4, P3, P4, O1, O2, F7, F8, T7, T8, P7, P8, F9, F10, FT9, FT10, TP9, TP10, P9, P10, PO9, PO10, I1, I2, with a 512-Hz sample rate from DC to 155 Hz. Please note that BioSemi systems work with a “zero-Ref” set-up with ground and reference electrodes replaced by a CMS/DRL circuit (for further information, see www.biosemi.com/faq/cms&drl.htm).

Contributions of blink artifacts were corrected using the algorithm implemented in BESA 5.1 (MEGIS Software GmbH, Graefelfing, Germany). EEG was segmented from −200 until 1000 ms relative to stimulus onset, with the first 200 ms as baseline. Trials contaminated by non-ocular artifacts and saccades were rejected from further analysis. Artifact rejection was carried out using the BESA 5.1 tool, with an amplitude threshold of 100 μV, as well as a gradient criterion of 75 μV. Remaining trials were recalculated to average reference, digitally low-pass filtered at 40 Hz (12 db/oct, zero phase shift), and averaged according to the 16 experimental conditions.

In the resulting waveforms, mean amplitudes and peak latencies for N170 were determined at P9/P10 between 140 and 180 ms for young adults and between 155 and 195 ms for older adults. Mean amplitude for P2 was measured at the same sites between 200 and 300 ms for both younger and older adults. Statistical analyses were performed by calculating mixed-model analyses of variance (ANOVA), with degrees of freedom corrected according to the Greenhouse-Geisser procedure where appropriate.

## Results

### Response times

**Figure 2:**
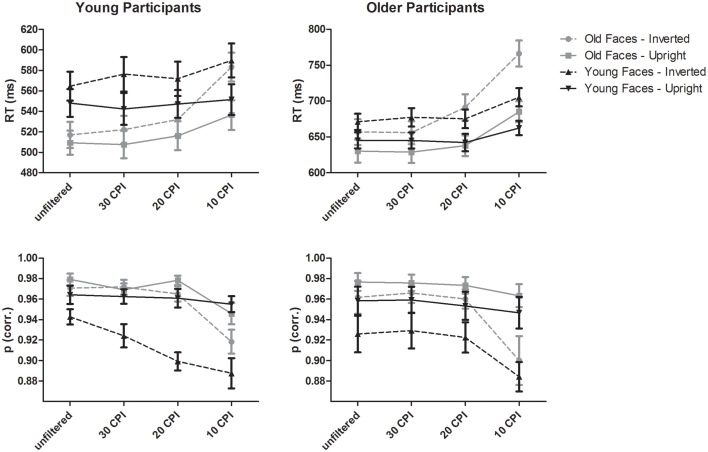
**Behavioral data from young and older participants**. Error bars depict standard errors of the mean.

A mixed-model ANOVA on mean response times (see upper part of Figure 2) with the within-subject factors face age (young, old), orientation (upright, inverted), filter (unfiltered, 30 CPI, 20 CPI, 10 CPI) and the between-subjects factor group (young adults, older adults) resulted in main effects of face age, *F*_(1, 46)_ = 5.97, *p* = 0.018, $\eta_{p}^{2}=$ 0.12, with faster responses for old as compared to young faces, orientation, *F*_(1, 46)_ = 212.23, *p* < 0.001, η^2^_*p*_ = 0.82, with slower RTs for inverted vs. upright faces, and filter, *F*_(3, 138)_ = 145.65, *p* < 0.001, $\eta_{p}^{2}=$ 0.76, indicating slower responses with increasing filter strength. As indicated by the effect of group, older adults responded slower than young adults, *F*_(1, 46)_ = 44.45, *p* < 0.001, $\eta_{p}^{2}=$ 0.49.

Most interestingly, interactions of face age by group, *F*_(3, 46)_ = 9.18, *p* = 0.004, $\eta_{p}^{2}=$ 0.17, and orientation by group, *F*_(1, 46)_ = 12.12, *p* = 0.001, $\eta_{p}^{2}=$ 0.21, were further qualified by a three-way interaction of face age by orientation by group, *F*_(1, 46)_ = 5.553, *p* = 0.023, $\eta_{p}^{2}=$ 0.11. *Post-hoc* tests in younger adults indicated a significant interaction of face age by orientation, *F*_(1, 23)_ = 4.49, *p* = 0.045, $\eta_{p}^{2}=$ 0.16, with larger inversion effects for young relative to old faces. In older adults, a significant main effect of orientation, *F*_(1, 23)_ = 123.50, *p* < 0.001, $\eta_{p}^{2}=$ 0.84, but no significant interaction with face age, *F*_(1, 23)_ = 2.81, *p* = 0.107, $\eta_{p}^{2}=$ 0.11, indicated statistically similar inversion effects for young and old faces.

An interaction of filter by group, *F*_(3, 138)_ = 12.56, *p* < 0.001, $\eta_{p}^{2}=$ 0.21, indicated similar response times in young adults for unfiltered vs. 30 CPI images, *F* < 1, but progressively slower response times for 30 vs. 20 CPI faces, *F*_(1, 23)_ = 5.03, *p* = 0.035, $\eta_{p}^{2}=$ 0.18, and 20 vs. 10 CPI faces, *F*_(1, 23)_ = 126.02, *p* < 0.001, $\eta_{p}^{2}=$ 0.85. Similar, in older adults response times for the unfiltered vs. 30 CPI conditions were similar, *F* < 1, whereas slower responses were observed in the 20 relative to the 30 CPI conditions, *F*_(1, 23)_ = 12.86, *p* = 0.002, $\eta_{p}^{2}=$ 0.36, and in the 10 relative to the 20 CPI conditions, *F*_(1, 23)_ = 89.44, *p* < 0.001, $\eta_{p}^{2}=$ 0.80. Please note that the interaction with group is probably due to the larger filter effect in older relative to younger adults from 30 to 20 CPI.

Finally, interactions of face age by filter, *F*_(3, 138)_ = 34.74, *p* < 0.001, $\eta_{p}^{2}=$ 0.43, and orientation by filter, *F*_(3, 138)_ = 24.31, *p* < 0.001, $\eta_{p}^{2}=$ 0.35, were further qualified by a three-way interaction of face age by orientation by filter, *F*_(3, 138)_ = 6.69, *p* = 0.001, $\eta_{p}^{2}=$ 0.127. *Post-hoc* tests (see Table 1) indicated similar response times for young upright faces in the unfiltered vs. 30 CPI, and in the 30 vs. 20 CPI conditions, but slower RTs in the 10 vs. 20 CPI conditions. Responses to inverted young faces were slower in the unfiltered relative to the 30 CPI condition, similar in the 30 vs. 20 CPI conditions, and slower in the 10 relative to the 20 CPI conditions. Response times for old upright faces were similar in the unfiltered relative to the 30 CPI condition, slower in the 20 relative to the 30 CPI condition, and further decreased in the 10 relative to the 20 CPI condition. Similarly, for old inverted faces response times were equivalent in the unfiltered relative to the 30 CPI condition, but slower in the 20 relative to the 30 CPI conditions, as well as in the 10 relative to the 20 CPI conditions.

**Table 1:** ***Post-hoc* tests of the interaction of face by orientation by filter in the analysis of response times**.

	**Unfiltered vs. 30 CPI**	**30 CPI vs. 20 CPI**	**20 CPI vs. 10 CPI**
**YOUNG FACES—UPRIGHT**			
*F*_(1, 47)_	< 1	< 1	9.41
*p*			0.004
$\eta_{p}^{2}$			0.17
**YOUNG FACES—INVERTED**			
*F*_(1, 47)_	4.47	< 1	20.35
*p*	0.040		< 0.001
$\eta_{p}^{2}$	0.09		0.30
**OLD FACES—UPRIGHT**			
*F*_(1, 47)_	< 1	4.66	63.85
*p*		0.036	< 0.001
$\eta_{p}^{2}$		0.09	0.58
**OLD FACES—INVERTED**			
*F*_(1, 47)_	< 1	29.46	115.04
*p*		< 0.001	< 0.001
$\eta_{p}^{2}$		0.39	0.71

In sum, analysis of response times revealed effects of low-pass filtering the images over and above the effects of face inversion, which were particularly pronounced for old faces in the strongest filter condition. Moreover, in young adults, inversion effects were stronger for young relative to old faces, whereas no differential inversion effect for young vs. old faces was detected in older participants.

### Accuracies

A mixed-model ANOVA on accuracies with the within-subject factors face age, orientation, and filter, and the between-subjects factor group revealed a main effect of face age, *F*_(1, 46)_ = 11.32, *p* = 0.002, $\eta_{p}^{2}=$ 0.20, with more correct responses to older compared to younger faces. Furthermore, upright as compared to inverted faces were more frequently correctly categorized, as indicated by the effect of orientation, *F*_(1, 46)_ = 141.92, *p* < 0.001, $\eta_{p}^{2}=$ 0.76. The main effect of filter, *F*_(3, 138)_ = 37.10, *p* < 0.001, $\eta_{p}^{2}=$ 0.47, revealed less accurate categorizations with increasing filter strength.

In addition, several interactions were found. Most interestingly, face age interacted with orientation, *F*_(1, 46)_ = 16.02, *p* < 0.001, $\eta_{p}^{2}=$ 0.26, and separate *post-hoc* ANOVAs for young and old faces revealed that the inversion effect was stronger for young, *F*_(1, 47)_ = 118.42, *p* < 0.001, $\eta_{p}^{2}=$ 0.72, than for old faces, *F*_(1, 47)_ = 19.94, *p* < 0.001, $\eta_{p}^{2}=$ 0.30. However, only a trend for an interaction of face age by orientation by group was observed, *F*_(1, 46)_ = 3.23, *p* = 0.079, $\eta_{p}^{2}=$ 0.07.

Furthermore, orientation interacted with filter, *F*_(3, 138)_ = 20.96, *p* < 0.001, $\eta_{p}^{2}=$ 0.31, which was further qualified by the group factor, *F*_(3, 138)_ = 3.18, *p* = 0.026, $\eta_{p}^{2}=$ 0.07. In young adults, *post-hoc* tests (see Table 2) for upright faces revealed less accurate responses in the 10 relative to the 20 CPI condition only. Correct responses for inverted faces were less frequent in the 20 relative to 30 CPI condition, as well as in the 20 relative to 10 CPI condition. In older adults, less accurate responses for upright faces were detected in the 10 relative to the 20 CPI condition only. For inverted faces, less accurate responses were detected in the 20 relative to the 10 CPI condition. No main effect of group was detected in accuracies, *F* < 1.

**Table 2:** ***Post-hoc* tests for the interaction of orientation by filter by group in the analysis of accuracies**.

	**Unfiltered vs. 30 CPI**	**30 CPI vs. 20 CPI**	**20 CPI vs. 10 CPI**
**YOUNG PART.—UPRIGHT**			
*F*_(1, 23)_	1.57	< 1	19.97
*p*	0.223		< 0.001
$\eta_{p}^{2}$	0.06		0.47
**YOUNG PART.—INVERTED**			
*F*_(1, 23)_	2.45	17.79	32.77
*p*	0.131	< 0.001	< 0.001
$\eta_{p}^{2}$	0.10	0.43	0.59
**OLDER PART.—UPRIGHT**			
*F*_(1, 23)_	< 1	1.63	6.37
*p*		0.215	0.019
$\eta_{p}^{2}$		0.06	0.22
**OLDER PART.—INVERTED**			
*F*_(1, 23)_	< 1	< 1	42.33
*p*			< 0.001
$\eta_{p}^{2}$			0.65

Overall, analysis of accuracy data suggested detrimental effects of low-pass filtering the images on age categorization over and above the effect of face inversion, particularly for the strongest filter condition (filtering frequencies higher than 10 CPI). Moreover, the face age by orientation interaction suggested more pronounced processing of relatively distant information for young relative to older faces for both young and old participants.

### Event-related potentials

**Figure 3:**
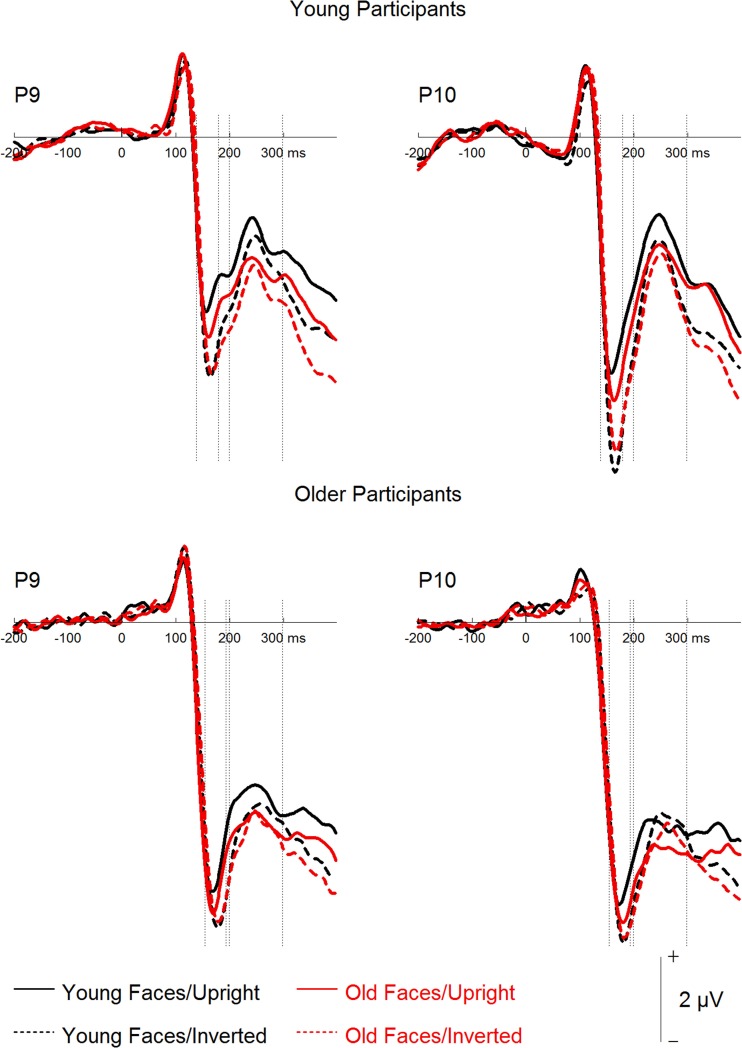
**Grand mean event-related potentials depicting the factors face age and orientation for young and older participants**.

**Figure 4:**
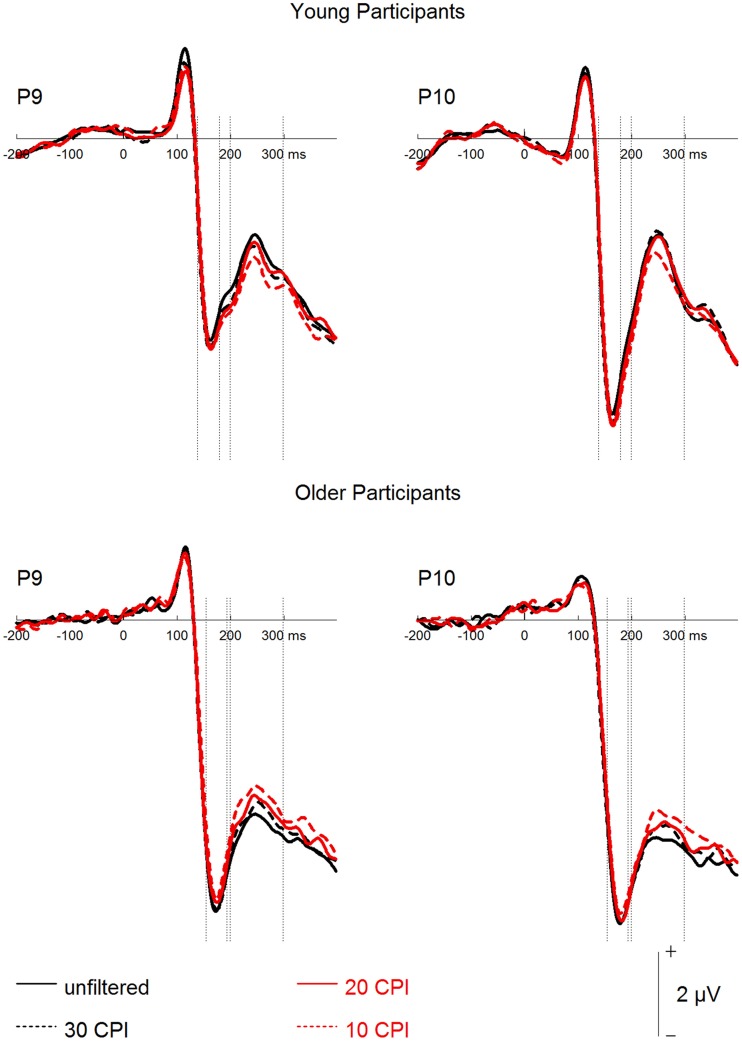
**Grand mean event-related potentials depicting the filter factor for young and older participants**.

A mixed-model ANOVA on N170 mean amplitudes (see Figures 3, 4) with the within-subject factors hemisphere (left, right), face age, orientation, and filter, and the between-subjects factor group resulted in effects of orientation, *F*_(1, 46)_ = 21.87, *p* < 0.001, $\eta_{p}^{2}=$ 0.32, with more negative amplitudes for inverted as compared to upright faces, and filter, *F*_(3, 138)_ = 4.02, *p* = 0.009, $\eta_{p}^{2}=$ 0.08, with less negative amplitudes for increasing filter strength. *Post-hoc* tests revealed no difference for the unfiltered vs. the 30 CPI condition, *F* < 1, and for the 30 compared to the 20 CPI condition, *F* < 1, but significantly less negative amplitudes in the 10 compared to the 20 CPI condition, *F*_(1, 47)_ = 7.08, *p* = 0.011, $\eta_{p}^{2}=$ 0.13. N170 amplitudes differed significantly between age groups, *F*_(1, 46)_ = 4.40, *p* = 0.042, $\eta_{p}^{2}=$ 0.09, with more negative amplitudes for older relative to younger adults. We further detected a trend for an interaction of orientation × group, *F*_(1, 46)_ = 3.48, *p* = 0.069, $\eta_{p}^{2}=$ 0.07, pointing toward larger inversion effects in the young as compared to the older group. Interestingly, orientation interacted with face age, *F*_(1, 46)_ = 43.43, *p* < 0.001, $\eta_{p}^{2}=$ 0.49. *Post-hoc* analyses for young and older faces separately revealed that inverted young faces elicited significantly more negative amplitudes than upright young faces, *F*_(1, 47)_ = 34.17, *p* < 0.001, $\eta_{p}^{2}=$ 0.42, whereas the corresponding pattern was not significant for old faces, *F*_(1, 47)_ = 3.27, *p* = 0.077, $\eta_{p}^{2}=$ 0.065. The interaction of hemisphere by group was not significant, *F*_(1, 46)_ = 2.287, *p* = 0.137, $\eta_{p}^{2}=$ 0.08. Moreover, no interaction of orientation by face age by group was observed, *F*_(1, 46)_ = 2.01, *p* = 0.163, $\eta_{p}^{2}=$ 0.04.

In sum, only a trend toward larger inversion effects in younger relative to older adults was detected. Moreover, and in line with the behavioral results, only small and non-significant inversion effects were found for old faces, and this was the case both for young and older adults.

**Figure 5:**
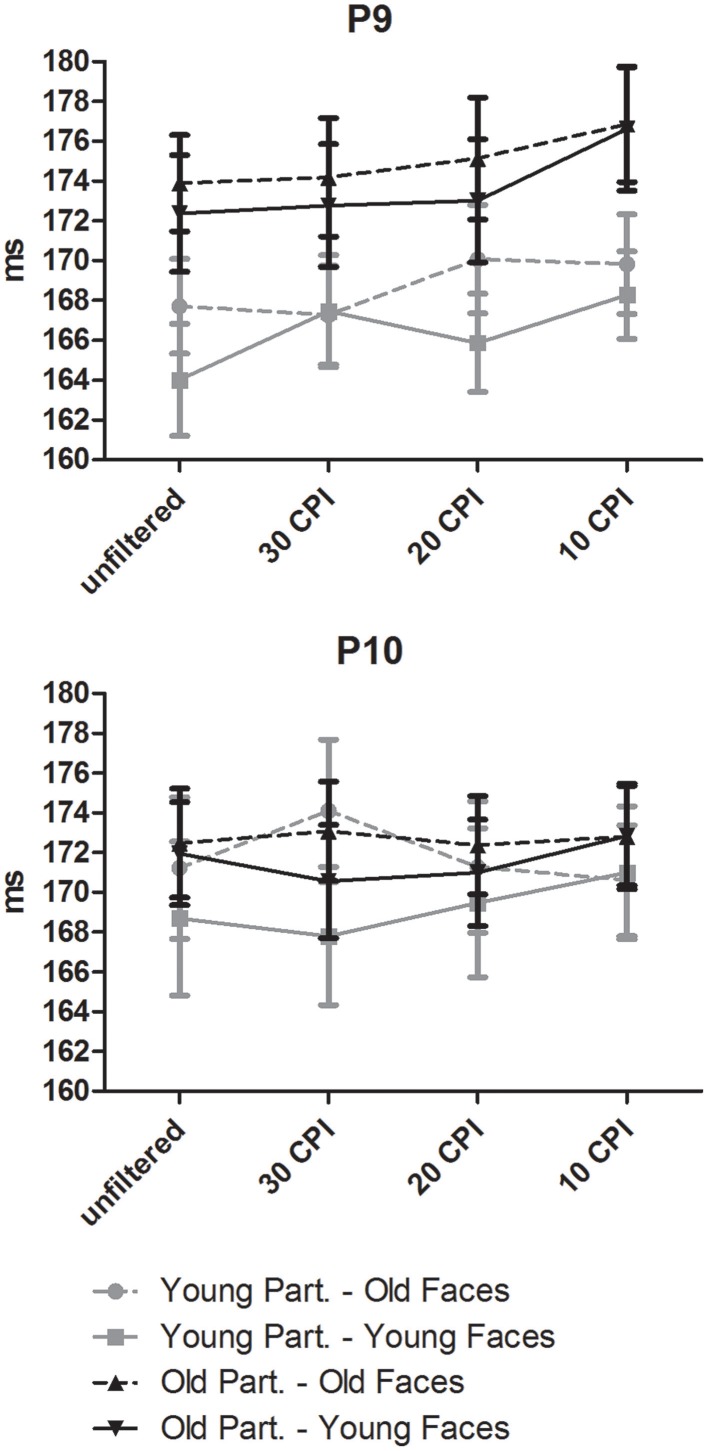
**Effects of face age, low-pass filtering, and participant age on mean N170 latency**. Error bars depict standard errors of the mean.

A mixed-model ANOVA on N170 peak latency with the within-subject factors hemisphere, face age, orientation, and filter, and the between-subjects factor group resulted in a main effect of face age, *F*_(1, 46)_ = 13.44, *p* = 0.001, $\eta_{p}^{2}=$ 0.23, with longer latencies for old than young faces. Moreover, inverted faces elicited longer latencies than upright faces, as indicated by the significant effect of orientation, *F*_(1, 46)_ = 47.77, *p* < 0.001, $\eta_{p}^{2}=$ 0.51. The filter factor reached significance, *F*_(1, 46)_ = 4.01, *p* = 0.009, $\eta_{p}^{2}=$ 0.08, indicating longer latencies for increased filtering strength. Furthermore, a three-way interaction of face age, hemisphere, and filter was detected, *F*_(3, 138)_ = 3.22, *p* = 0.025, $\eta_{p}^{2}=$ 0.65. Separate analyses for the left and the right hemisphere and for old and young faces (see Figure 5) indicated the absence of a filter effect over the right hemisphere, both for young faces, *F*_(3, 141)_ = 2.12, *p* = 0.101, $\eta_{p}^{2}=$ 0.43, and for old faces, *F*_(3, 141)_ = 1.19, *p* = 0.315, $\eta_{p}^{2}=$ 0.03. By contrast, over the left hemisphere the filter effect reached significance for both young, *F*_(3, 141)_ = 5.13, *p* = 0.002, $\eta_{p}^{2}=$ 0.10, and old faces, *F*_(3, 141)_ = 3.07, *p* = 0.030, $\eta_{p}^{2}=$ 0.06, and was somewhat more pronounced in the former case. Planned comparisons did not reveal any significant differences between filter conditions for young faces; unfiltered vs. 30 CPI: *F* < 1, 30 vs. 20 CPI: *F*_(1, 47)_ = 2.90, *p* = 0.097, $\eta_{p}^{2}=$ 0.06, 20 vs. 10 CPI: *F* < 1. By contrast, for older faces the unfiltered condition did not differ from the 30 CPI condition, *F*_(1, 47)_ = 2.53, *p* = 0.12, $\eta_{p}^{2}=$ 0.05, and the 30 CPI did not differ from the 20 CPI condition, *F* < 1. However, filtering images at 10 CPI resulted in a delayed N170 peak relative to the 20 CPI condition, *F*_(1, 47)_ = 7.44, *p* = 0.009, $\eta_{p}^{2}=$ 0.14. The group factor did not reach significance, *F*_(1, 46)_ = 1.72, *p* = 0.20, $\eta_{p}^{2}=$ 0.04. Similarly, no interaction of orientation by face age by group was observed, *F* < 1. In sum, both inversion and low-pass filtering resulted in delayed N170 peaks. The filter effect, however, was small and restricted to the strongest condition, old faces and the left hemispheric electrode site.

In order to test for a direct relationship between inversion effects observed in RTs and N170 measured at P10, correlations between the difference of inverted and upright faces in both measures were calculated. This analysis revealed a significant relationship between the two measures when all participants were entered into the analysis, *r* = 0.395, *p* = 0.005, but neither for young (*r* = 0.310, *p* = 0.141) nor older adults separately (*r* = 0.323, *p* = 0.124). Similarly, a corresponding analysis using N170 latency differences at P10 did not result in significant effects (all *r* < 0.181 and > −0.223, all *p* > 0.295).

Finally, a mixed-model ANOVA on P2 mean amplitudes with the within-subject factors hemisphere, face age, orientation, and filter, and the between-subjects factor group revealed main effects of face age, *F*_(1, 46)_ = 48.93, *p* < 0.001, $\eta_{p}^{2}=$ 0.52, with more positive amplitudes for young relative to old faces, and orientation, *F*_(1, 46)_ = 13.66, *p* = 0.001, $\eta_{p}^{2}=$ 0.23, with more positive amplitudes for upright than inverted faces. The group factor interacted with filter, *F*_(3, 138)_ = 16.34, *p* < 0.001, $\eta_{p}^{2}=$ 0.26. Subsequent analyses for the two groups separately (see Figure 4) revealed filter effects for both older, *F*_(3, 69)_ = 11.45, *p* < 0.001, $\eta_{p}^{2}=$ 0.33, and young participants, *F*_(3, 69)_ = 6.17, *p* = 0.001, $\eta_{p}^{2}=$ 0.21. In the older group, however, increasing filter strength elicited more positive amplitudes [unfiltered vs. 30 CPI: *F*_(1, 23)_ = 6.12, *p* = 0.021, $\eta_{p}^{2}=$ 0.21; 30 vs. 20 CPI: *F*_(1, 23)_ = 2.97, *p* = 0.098, $\eta_{p}^{2}=$ 0.11; 20 vs. 10 CPI: *F*_(1, 23)_ = 6.09, *p* = 0.021, $\eta_{p}^{2}=$ 0.21], whereas in the younger group increasing filter strength elicited less positive amplitudes [unfiltered vs. 30 CPI: *F* < 1; 30 vs. 20 CPI: *F* < 1; 20 vs. 10 CPI: *F*_(1, 23)_ = 8.85, *p* = 0.007, $\eta_{p}^{2}=$ 0.28]. In addition, face age interacted with orientation, *F*_(1, 46)_ = 5.73, *p* = 0.021, $\eta_{p}^{2}=$ 0.11. *Post-hoc* tests for younger and older faces separately (see Figure 3) resulted in an orientation effect for both face age conditions, which was, however, more pronounced for young, *F*_(1, 46)_ = 19.62, *p* < 0.001, $\eta_{p}^{2}=$ 0.30, relative to old faces, *F*_(1, 46)_ = 6.02, *p* = 0.018, $\eta_{p}^{2}=$ 0.12. No significant interaction of face age by orientation by group was observed, *F* < 1.

Overall, similar to the analyses of our behavioral data and N170 amplitude, inversion effects were more pronounced for young relative to old faces. In addition, low-pass filtering resulted in less positive amplitudes in young adults, but more positive amplitudes in older participants.

### Comparisons within the older participant group

As the match between face and participant age was closer for young relative to older participants, we calculated additional analyses within the older participant group. For that purpose, we conducted a median split in our older group based on age, which resulted in a young older adult (YOA) and an old older adult (OOA) group (*N* = 12 per group; YOA mean age = 62 years ± 2 SD; OOA mean age = 70 years ± 2 SD)^1^.

A mixed-model ANOVA on RTs (see Table 3) with group (YOA, OOA) as a between-subjects factor and face age, orientation and filter as within-subject factors revealed no significant interaction of face age by orientation by group, *F*_(1, 22)_ = 1.94, *p* = 0.178, $\eta_{p}^{2}=$ 0.081, and none of the other interactions with group resulted in significant effects. A corresponding ANOVA on accuracies (see Table 3) yielded a significant interaction of orientation by group, *F*_(1, 22)_ = 4.50, *p* = 0.045, $\eta_{p}^{2}=$ 0.170, with larger inversion effects in the OOA group. Again, the interaction of face age by orientation by group was not significant, *F*_(1, 22)_ = 2.24, *p* = 0.149, $\eta_{p}^{2}=$ 0.092. No further effects involving the group factor were significant.

**Table 3:** **Response times and accuracies (means and standard errors of the means) for Young Older and Old Older participants**.

	**Young older adults**								**Old older adults**							
	**Unfiltered**		**30 CPI**		**20 CPI**		**10 CPI**		**Unfiltered**		**30 CPI**		**20 CPI**		**10 CPI**	
**RESPONSE TIMES**																
Young Faces—Upright	649.14	14.30	646.40	15.18	650.18	16.76	674.51	16.11	549.44	19.52	538.36	20.08	536.87	15.10	543.93	19.34
Young Faces—Inverted	671.74	19.70	679.75	18.88	677.06	18.22	720.41	17.60	560.18	18.02	566.61	18.17	567.83	22.43	585.04	22.26
Old Faces—Upright	637.59	23.00	630.95	21.42	644.84	22.58	694.72	22.22	505.97	13.89	502.55	13.02	510.85	13.20	540.25	15.65
Old Faces—Inverted	658.42	24.58	649.15	21.35	680.07	20.86	759.26	25.60	520.66	16.35	521.74	14.20	529.42	13.62	583.97	17.59
**ACCURACIES**																
Young Faces—Upright	0.97	0.02	0.96	0.02	0.96	0.02	0.94	0.02	0.95	0.02	0.96	0.02	0.94	0.02	0.95	0.02
Young Faces—Inverted	0.95	0.02	0.93	0.03	0.93	0.02	0.88	0.02	0.91	0.03	0.93	0.02	0.92	0.02	0.89	0.02
Old Faces—Upright	0.98	0.01	0.97	0.01	0.98	0.01	0.96	0.01	0.98	0.01	0.98	0.01	0.97	0.01	0.96	0.02
Old Faces—Inverted	0.98	0.01	0.97	0.01	0.97	0.01	0.93	0.02	0.95	0.03	0.96	0.02	0.95	0.01	0.87	0.04

**Figure 6:**
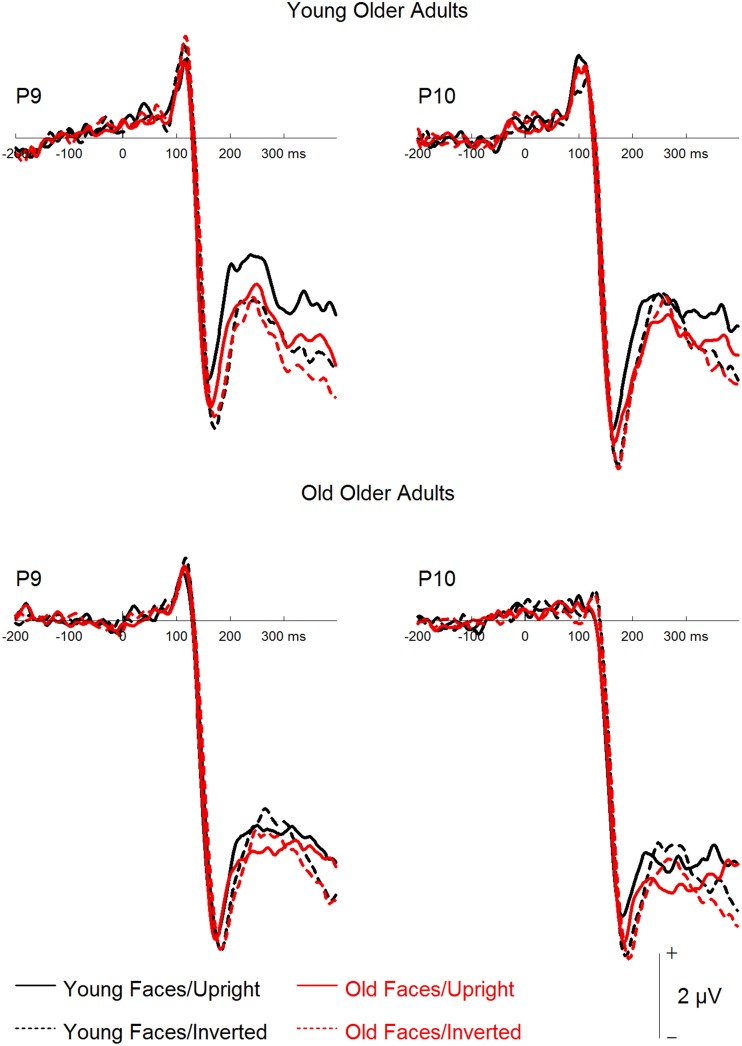
**Grand mean event-related potentials depicting the factors face age and orientation for young older adults and old older adults**.

An ANOVA on N170 amplitude (see Figure 6) with an additional within-subjects factor hemisphere revealed a significant interaction of orientation by group, *F*_(1, 22)_ = 7.80, *p* = 0.011, $\eta_{p}^{2}=$ 0.262, reflecting significant inversion effects in the YOA group, *F*_(1, 11)_ = 12.39, *p* = 0.005, $\eta_{p}^{2}=$ 0.530, but not in the OOA group, *F* < 1. Furthermore, a trend toward a significant interaction of face age by orientation by group was detected, *F*_(1, 22)_ = 3.52, *p* = 0.074, $\eta_{p}^{2}=$ 0.138. While both groups showed larger inversion effects for young relative to old faces, this pattern appeared less pronounced in the OOA group. No further effects involving the group factor were significant (all *p* > 0.1). A corresponding analysis on N170 peak latency revealed a trend toward a main effect of group, *F*_(1, 22)_ = 3.53, *p* = 0.074, $\eta_{p}^{2}=$ 0.138, with numerically longer N170 latencies in the OOA relative to the YOA group, and a significant interaction of face age by group, *F*_(1, 22)_ = 6.50, *p* = 0.018, $\eta_{p}^{2}=$ 0.228, with longer latencies for old relative to young faces in the YOA group, but no respective difference in the OOA group. The interaction of face age by orientation by group was not significant, *F*_(1, 22)_ = 1.90, *p* = 0.182, $\eta_{p}^{2}=$ 0.080. No additional effects involving the group factor were detected (all *p* > 0.1).

Finally, a corresponding mixed-model ANOVA on P2 amplitude yielded a trend toward an interaction of face age by orientation by group, *F*_(1, 22)_ = 3.00, *p* = 0.097, $\eta_{p}^{2}=$ 0.120, with larger inversion effects for young relative to old faces in the YOA group and no clear inversion effects in the OOA group. None of the other effects involving the group factor were significant.

In sum, the analyses reported in this section did not detect strong hints for a processing advantage for old faces in the OOA group. It should be noted, however, that the sample size might have been too small to detect subtle effects, and therefore the absence of significant effects should be treated with caution.

## Discussion

The present study examined the categorization of young and old faces according to age in young and older adult participants. We were particularly interested to examine (1) whether and to what extent the simultaneous processing of information from relatively distant parts of the face and more local texture-based information contribute to the perception of facial age, (2) whether old and young faces are perceived similarly, and whether the perception of facial age is biased by participant age, and (3) whether older adults would be less efficient in early face perception, and more specifically whether they would show reduced N170 inversion effects. The following paragraphs discuss these questions on the basis of the present findings and the previous literature.

### Both relatively distant and local information contribute to efficient age categorization

Our behavioral and ERP data suggest that efficient age categorization depends on both relatively distant and local information. This interpretation is based on the finding that both inversion and low-pass filtering resulted in slower and less accurate responses. More interestingly, the two manipulations interacted, as revealed by the more pronounced costs of low-pass filtering for inverted relative to upright faces. It thus seems that a narrowing of the perceptual field in inverted faces can be partly compensated by using local texture-based information, such that if this information is additionally removed, additional costs apply. The stepwise filtering approach used in the present study, with four increasingly severe cut-off frequencies, allowed pinning down the frequency range most informative for this partial compensation to between 20 and 10 CPI. The finding of reduced response times for inverted faces is in line with a previous report of an inversion effect in age categorization (Wiese et al., 2012a). At the same time, neither inversion nor filter effects were found in a study, in which the exact age of the face stimuli had to be estimated (George and Hole, 2000). Together with the present results, these previous findings indicate that the processing of facial age, although not impossible for inverted and low-pass filtered images, is substantially reduced in efficiency.

Generally in line with the behavioral results discussed above, both face inversion and low-pass filtering affected N170 and P2. Also in parallel to performance measures, filtering effects were largely restricted to the most severe 10 CPI cut-off frequency filter condition. Interestingly, the two factors did not interact. At first sight it appears plausible to assume that an inversion effect independent of filtering and a filter effect that is evident only in the strongest cut-frequency condition add up to the interaction observed in the behavioral data. It should be noted, however, that inversion and filter effects go in opposite directions: in line with previous studies (e.g., Rossion et al., 2000; Goffaux et al., 2003), we observed larger N170 amplitudes for inverted faces and smaller amplitudes for severely low-pass filtered faces. This seemingly contradictory finding is reminiscent of the long-known apparent paradox that the N170 is larger for upright faces relative to objects, while it is at the same time smaller for upright relative to inverted faces (Itier et al., 2006; Eimer, 2011). It thus seems that the effect of low-pass filtering is related to the former effect of generic face sensitivity, and that blurring may make the faces appear less face-like, probably because the first-order configuration of facial features is harder to detect. The finding that N170 is similarly reduced when high frequency noise is added to the image (Jemel et al., 2003) additionally supports this interpretation. Overall, relative to the N170 for unfiltered upright faces, both reduced and enhanced amplitudes seem to hamper the efficiency of age categorization.

### Relatively distant information is less important for categorizing old faces, but age categorization is not modulated by viewers' age

A further interesting finding of the present study was that young and old faces were processed differently to some extent. While old faces were categorized generally faster, inversion effects were more pronounced for young faces in both accuracies and ERPs, indicating less processing of relatively distant information for old faces. Moreover, when simultaneous processing of relatively distant information was possible, i.e., when images were presented in upright orientation, low-pass filtering affected response times for young faces only in the strongest filter condition, whereas filtering frequencies higher than 20 CPI affected the categorization of old faces. It thus seems that frequencies between 30 and 20 CPI contribute to the efficient categorization of old but not young faces, which are more robust against low-pass filtering. At the same time, when processing relatively distant information was disrupted by face inversion, high frequency information was informative for the detection of young age, as indicated by a decrease in response times starting already in the 30 CPI condition. This was not the case for old faces, which showed similar patterns of response time decrease with stronger filtering in the upright and inverted conditions. It thus seems that different frequency bands are drawn on when categorizing young and old faces if information from relatively distant parts of the face cannot be used.

At the same time, these results suggest that categorizing young faces predominantly depends on information from relatively distant parts and low-frequency information. Accordingly, only strong low-pass filtering affects the categorization of upright young faces. However, if the former type of information is not available, a more demanding analysis of local texture is conducted, which more strongly depends on higher frequency information, and is therefore hampered by even moderate low-pass filtering. Categorizing older faces depends on the processing of relatively distant information to a lesser extent, which is reflected in relatively smaller inversion effects. The relatively stronger use of local texture information for old faces is further reflected in the similar effects of low-pass filtering for upright and inverted faces.

This interpretation is at least partly supported by the ERP data: Parallel to the accuracy results, clearly larger inversion effects for young relative to old faces were observed in N170 and P2 amplitude measures. Accordingly, old faces are processed more similar when seen in upright vs. inverted orientation than young faces, and these different inversion effects might reflect differential processing of first- (N170) and second-order configural information (P2; see Latinus and Taylor, 2006). Again, it appears that processing of relatively distant information is more pronounced for young relative to old faces. At the same time, the effect of high-pass filtering the face images on N170 amplitude was similar for young and old faces. Accordingly, inverting and low-pass filtering the images appear to affect independent processes that contribute to N170 amplitude, with the former being sensitive to face age whereas the latter is not. Moreover, a strong low-pass filter affected the N170 latency for old faces but not young faces. This finding is broadly in line with the somewhat stronger sensitivity of old faces to the filter manipulation in response times. It should be noted, however, that the exact pattern found in response times is not paralleled in N170 latency results. Whereas, response times reflect the outcome of a cascade of sub-processes, including perceptual and decisional stages, N170 represents a more specific measure of early face perception. Therefore, it seems that later processing stages not reflected in our ERP analysis additionally modulated the pattern of results observed in response times.

As stated in the introduction, the own-race face recognition bias is at least partly based on more efficient perceptual processing of facial information (for a recent review, see Hayward et al., 2013). A neural correlate of this is seen in larger N170 amplitudes for other-race (e.g., Wiese et al., 2014) and larger N170 inversion effects for own-race faces (Vizioli et al., 2010; Caharel et al., 2011; Wiese, 2013). In the present study, we were interested in whether the previously described own-age recognition bias in young adults (Rhodes and Anastasi, 2012; Wiese et al., 2013b) was similarly paralleled by differences at early perceptual processing stages. The present results revealed only moderate evidence for this idea. On the one hand, young faces elicited larger RT inversion effects than old faces in young adults, whereas no differential inversion effect was observed in older adults. On the other hand, both N170 and P2 inversion effects were larger for young faces, and this effect did not interact with participant age. Similarly, in a previous study we observed a larger N170 misalignment effect (with larger amplitudes for horizontally misaligned relative to aligned face halves) for young relative to old faces in both young and older adults (Wiese et al., 2013a). Overall, assuming that the present young participants would show an own-age bias in face memory if so tested, the present results do not provide strong evidence for an early perceptual basis of this own-age memory bias. Our data instead suggest that the processing of relatively distant information is less important for old faces for both young and older adults. The absence of a stronger inversion effect for young faces in older adults in one out of four measures can be hardly interpreted as a strong argument against this suggestion.

As a potential limitation of the present study, we note that the match between stimulus and participant age was closer in young relative to older adults, and that the absence of a processing advantage for old faces in older adults might be partly related to this larger mismatch. It should be noted, however, that at least the own-age bias in adult participants' recognition memory does not depend on an exact match of stimulus and participant age (Wolff et al., 2012). Moreover, previous studies have demonstrated that the age of older faces is particularly hard to perceive (George and Hole, 2000; Voelkle et al., 2012), probably because differences in neurobiological and socio-environmental factors (such as sun exposure, smoking etc.) have more time to affect facial appearance with increasing age. Interestingly, these studies have further shown that the age of older adults' faces is systematically underestimated by 4–5 years. This indicates that even though the face images in the present study were de facto older than the OOA participants, this has likely not been perceived as clearly as suggested by the difference in chronological age. Nevertheless, future studies should be stricter when matching stimulus and participant age for older participants.

### Older adults are overall less efficient in early face perception, but process young and old faces as younger adults

Although a number of differences between participant groups were detected in the present study, it appears important to point out that the results revealed only moderate effects of participant age on both behavioral and ERP data. Analysis of accuracy data hinted toward a slightly higher sensitivity to low-pass filtering facial information in younger adults. More precisely, when processing of relatively distant information was disrupted (i.e., in the inverted condition), a cut-off frequency at 20 CPI led to less accurate categorizations in younger but not older adults. This finding may indicate a somewhat stronger sensitivity to high frequency information in younger adults when information from relatively distant face parts cannot be used. It should be noted that this interpretation implies a link between configural and spatial frequency information, which has been found in some (Goffaux and Rossion, 2006), but not all studies (Boutet et al., 2003; Gaspar et al., 2008) examining this potential relationship in identity judgment tasks. Such subtle effects may also be related to slight differences in visual acuity between groups, which were not explicitly tested in the present study. Although all participants reported normal vision and wore their seeing aids if necessary, previous studies have shown that age group differences in vision remain even under these circumstances (see e.g., Komes et al., 2014a). Moreover, in the present study the overall pattern of response time decreases with increasing filter strength was similar for both age groups, suggesting only moderate age-related change in the present task.

In line with slowing accounts of cognitive aging (e.g., Salthouse, 1996), older participants needed more time for age categorizations than younger adults. If slower age categorization were linked to slowed perceptual processing, and given that N170 reflects a perceptual processing stage (such as the processing of first-order configuration or structural encoding), one might assume that its peak would be substantially delayed in older adults. The present data, however, do not point toward a perceptual locus of this effect, as N170 latency was not significantly delayed in older adults. As a potential qualification it should be noted that some previous studies observed delayed N170 peaks with increasing age (Gazzaley et al., 2008; Wolff et al., 2012), and that a trend in this direction was observed in the comparison of relatively young and old older adults in the present study.

N170 was larger for older adults, a finding that replicated previous results from others (Gao et al., 2009; Daniel and Bentin, 2012) and our group (Wiese et al., 2008; Wolff et al., 2012). More interestingly, and similar to previous studies, the N170 inversion effect was more pronounced in younger relative to older adults (Gao et al., 2009; Daniel and Bentin, 2012), although in the present study the respective interaction was only observed as a statistical trend. However, the analysis of young vs. old older adults yielded smaller N170 inversion effects in the latter group, suggesting that this age-related change in neural processing occurred after the age of 62. Importantly, this effect was observed even though young and old face stimuli were used. This finding suggests that reduced inversion effects reported in previous studies presumably reflected moderate but clearly detectable age-related changes in neural correlates of face perception rather than an experience-based bias toward own-age faces in the younger participants.

Interestingly, the larger N170 for inverted relative to upright faces has been suggested to reflect the recruitment of additional neural mechanisms (related to feature-based object processing or processing eyes) rather than stronger activation of the neural mechanism for upright faces (Rossion and Gauthier, 2002; Itier et al., 2006; Sadeh and Yovel, 2010). Thus, one might assume that the reduced N170 inversion effect in older adults indicates a deficit in this additional recruitment of processes associated with analyzing more local information for inverted faces. However, in the present study the reduced inversion effect in older adults was at least partly related to more negative N170 amplitudes for upright faces (mean amplitude in young adults: −4.4 μV; older adults: −6.2 μV), and not to the same extent to age-related differences for inverted faces (young adults: −5.2 μV; older adults: −6.6 μV). Given that the upright N170 reflects the simultaneous processing of relatively distant information, this finding may be interpreted as reflecting more effort and thus reduced efficiency for this type of processing. Moreover, the smaller increase in negativity from upright to inverted faces in older adults may reflect less recruitment of additional local processing.

At the same time, N170 effects of face age and filtering were similarly observed in younger and older adults. Whereas, the more negative N170 for old relative to young faces in both groups is generally in line with a previous study (Komes et al., 2014b), which also found that the fine-tuning of N170 to faces from different ethnic groups is largely intact in older age, the present findings further suggest that N170 sensitivity to information from different frequency bands also seems to be largely preserved in older participants. Assuming that the effect of low-pass filtering on N170 amplitude is related to the generic face sensitivity of this component, our findings are generally in line with the conclusion that this aspect of N170 is largely intact in older adults (Daniel and Bentin, 2012). Overall, together with previous ERP studies, the present results indicate selective age-related effects of face inversion on N170, which at least partly reflects less efficient processing of relatively distant information in older adults. At the same time, clear inversion effects were observed in both groups in the behavioral results of the present study, which again support the interpretation of only moderate age-related change.

A number of previous behavioral studies tested the simultaneous processing of relatively distant information in older adults. Interestingly, both Boutet and Faubert (2006), who tested the inversion and composite face effect, and Konar et al. (2013), who examined the composite face effect only, concluded that configural processing is not reduced in older adults. Of note, however, Boutet and Faubert (2006) did not analyze response times. Therefore, they might have missed aging effects manifesting in less efficient processing. Moreover, Konar et al. (2013) did observe an effect of aging on response times, which together with similar accuracies may be interpreted as less efficient processing (see also Wiese et al., 2013a). Finally, Hildebrandt et al. (2010) did not observe a composite effect in older participants and found that effect sizes for the inversion effect were substantially smaller for older relative to both middle-aged and young participants. In conclusion, and generally in line with previous and the present ERP results, it appears that some age-related changes in the simultaneous processing of information from relatively distant parts of faces (which is measured in “configural” tasks; see Rossion, 2009) are typically observed, either reflecting less accurate or, more subtly, less efficient processing in older adults. Accordingly, future research should take both accuracy and efficiency of processing into account.

It should be noted, however, that in the present study age-related differences were observed in the N170 but not in the behavioral data. Similar apparent discrepancies between ERP and behavior have been observed in other research areas (e.g., in language processing; see e.g., Federmeier and Kutas, 2005; Federmeier et al., 2010). In principle, we see two possible underlying causes: (1) a specific processing stage (in the present case the processes reflected by N170) is affected by aging, but this deficit is compensated at a later processing stage, or (2) ERPs are more sensitive to detect age-related changes than behavioral measures, and therefore point to changes that will manifest at the behavioral level in higher age. With respect to the first suggestion, we note that an increased recruitment of higher-order cognitive processes to compensate for age-related deficits in sensory and perceptual processes has been suggested by neuroimaging studies (see Dennis and Cabeza, 2008). However, as the present results do not provide direct evidence for such a compensation mechanism, this interpretation remains speculative. At the same time, the second suggestion is not in line with our finding of similar (or even larger) behavioral inversion effects in old older relative to young older adults. Further research is clearly needed to clarify the repeatedly observed mismatch with respect to age-related changes between ERP and behavioral data.

In addition, aging seems to clearly affect the neural processes reflected in ERP components following N170. In the present study, filtering face images had opposite effects on the P2 of younger and older adults (for potentially related findings in slightly later time windows, see Wiese et al., 2012b; Komes et al., 2014a). Whereas, younger adults demonstrated less positive amplitudes with increased filter settings, more positive amplitudes were observed in older adults. This finding may point to a differential orientation and/or location of the underlying generators (e.g., Jackson and Bolger, 2014) as a consequence of age-related brain changes. Alternatively, differences in processing strategies may account for this finding. The occipito-temporal P2 is larger in attentionally more demanding conditions (Neumann et al., 2015). One possibility to explain the above pattern is to assume that older adults tried to compensate for the increased difficulty in the filtered conditions by enhancing attentional resources. Irrespective of the precise underlying cause, ERPs in time ranges following N170 appear to be modulated in older relative to younger adults in a qualitatively different manner, whereas age-related modulations of the N170 are quantitative.

A potential qualification of the present results may lie in the repetition of facial identities (although the same identity was never presented twice in the same condition). This, together with the presentation time of 1 s, may have encouraged participants to not only process the directly task-relevant age information, but also task-irrelevant identity information. If so, this appears unlikely to have affected our main results. First, with respect to our behavioral findings, one might assume that with increasing face familiarity over blocks, configural processing of the faces might increase. This, however, was not the case. An additional ANOVA on RTs, with the within-subject factors block (five levels) and orientation, and a between-subjects factor group neither revealed a significant two-way interaction of block by orientation, *F*_(4, 184)_ = 1.34, *p* = 0.243, $\eta_{p}^{2}=$ 0.029, nor a three-way interaction of block × orientation × group interaction, *F*_(4, 184)_ = 1.51, *p* = 0.200, $\eta_{p}^{2}=$ 0.032. In addition, we note that several recent papers question the relationship between configural and identity processing (Taschereau-Dumouchel et al., 2010; Burton et al., 2015). However, independent of whether configural information is important for identity processing, a narrowing of the perceptual field, which may result from face inversion (Rossion, 2009), may slow down age categorizations, suggesting that the simultaneous processing of several relatively distant parts of the face is relevant for efficient age categorization. Second, with respect to our ERP findings we note that most researchers agree that the N170 reflects processes prior to the identification of individual faces (Bentin and Deouell, 2000; Eimer, 2000b; Schweinberger et al., 2002; Henson et al., 2003). Reports associating this component with identity processing typically show very small effects, and are inconsistent with respect to their direction (see Caharel et al., 2006; Marzi and Viggiano, 2007). It therefore appears unlikely that the present results in the N170 reflect the processing of identity rather than age information.

## Conclusions

The present study examined the interplay of two arguably fundamental age-related changes: the change in facial appearance and the change in perceptual and cognitive functioning with increasing age. We found that both information from relatively distant parts of the face and local information are used for the processing of facial age. Moreover, the simultaneous processing of information from distant parts seems to be relatively more important for perceiving young as compared to old faces. This effect was similarly observed in young and older participants, arguing against the idea of an own-age bias in young adults' early face perception. Finally, moderate effects of cognitive aging on face perception were detected in the present study, which is in line with previous research (Hildebrandt et al., 2010).

### Conflict of interest statement

The authors declare that the research was conducted in the absence of any commercial or financial relationships that could be construed as a potential conflict of interest.

## Footnotes

^1^Please note that the chronological age of the face stimuli was still significantly higher than participant age in the OOA group (Mann–Whitney *U* = 87.0, *p* < 0.001).
